# Perception of the relative harm of electronic cigarettes compared to cigarettes amongst US adults from 2013 to 2016: analysis of the Population Assessment of Tobacco and Health (PATH) study data

**DOI:** 10.1186/s12954-020-00410-2

**Published:** 2020-09-18

**Authors:** Layla Malt, Thomas Verron, Xavier Cahours, Mengran Guo, Sarah Weaver, Tanvir Walele, Grant O’Connell

**Affiliations:** 1 Imperial Brands Plc., 121 Winterstoke Road, Bristol, BS3 2LL UK; 2 SEITA, Imperial Brands, 143 Boulevard Romain Rolland, 75014 Paris, France

**Keywords:** E-cigarettes, Relative harm perception, Harm reduction, Public health, Tobacco, Cigarette

## Abstract

**Background:**

Electronic cigarettes (e-cigarettes) have been characterised as significantly less harmful than cigarettes by many health agencies and regulators globally. In this study, we examined to what extent perceived relative harms of e-cigarettes compared to cigarettes have changed in the USA.

**Methods:**

We analysed the data from the longitudinal and nationally representative, Population Assessment of Tobacco and Health Study to assess the relative perceived harm of e-cigarettes amongst US adults between 2013 and 2016.

**Results:**

The proportion of US adults who correctly perceived e-cigarettes as less harmful than cigarettes decreased each year from 41.1% (CI 40.1–42.1%) in 2013–2014, 31.5% (CI 30.8–32.2%) in 2014–2015 and 25.3% (CI 24.6–26.0%) in 2015–2016. Concurrently, the proportion of US adults who perceived e-cigarettes as equally, or more, harmful than cigarettes increased from 53.7% (CI 52.3–55.1%), 64.9% (CI 63.6–66.2%) to 72.7% (CI 71.5–73.9%) respectively. The proportion of US adults who held negative relative harm perceptions of e-cigarettes increased regardless of current smoking or vaping status by 24.6% and 29.6% respectively within 3 years. In Wave 3, the proportion of current smokers who perceived the relative harm of e-cigarettes as less harmful was lower at 29.3% (CI 28.2–30.4%) compared to current e-cigarette users at 43.5% (CI 40.3–46.7%). Former smokers who used e-cigarettes and believed that they were equally, or more, harmful than cigarettes in 2014–2015 had significantly higher rates of smoking relapse in the following year, 29% and 37% (*p* < 2.2e−16), respectively, compared to those with positive relative harm perceptions who reported relapse rates of 19%.

**Conclusions:**

In this study, the proportion of US adults who incorrectly perceived e-cigarettes as equal to, or more, harmful than cigarettes increased steadily regardless of smoking or vaping status. Current adult smokers appear to be poorly informed about the relative risks of e-cigarettes yet have potentially the most to gain from transitioning to these products. The findings of this study emphasise the urgent need to accurately communicate the reduced relative risk of e-cigarettes compared to continued cigarette smoking and clearly differentiate absolute and relative harms. Further research is required to elucidate why the relative harm of e-cigarettes is misunderstood and continues to deteriorate.

## Introduction

There are currently over 1 billion smokers in the world [1]. Over the last 50 years, public health authorities have worked to ensure that the harms associated with smoking are well understood. Despite these efforts, the World Health Organisation estimates that the number of smokers will increase to 1.1 billion by 2025 [2]. Whilst combustible cigarettes continue to remain the most commonly used nicotine product globally, the use of electronic cigarettes(s) (EC) is increasing worldwide by adult smokers who are seeking less harmful alternatives to combustible tobacco. There is scientific agreement that the most detrimental effects of smoking are due to the formation of toxicants from tobacco combustion and inhalation of tobacco smoke, and not by nicotine. According to the FDA, a key aspect of the organisation’s comprehensive plan for tobacco and nicotine regulation involves demonstrating a greater awareness that nicotine is delivered through products that represent a continuum of risk, and that delivery is most harmful when delivered through smoke particles in cigarettes [3]. The emergence, awareness and use of EC, which do not contain, nor combust, tobacco leaf has led to a transformation in the way adult smokers consume nicotine. This has subsequently triggered significant debate on the role of nicotine within society, beyond tobacco combustion, when decoupled from tobacco smoke.

As epidemiological data is limited, due to the relatively short time EC have been commercially available, the potential long-term health effects of EC are not yet fully understood. However, there is a growing global body of evidence which indicates EC are likely to be significantly less harmful compared to smoking tobacco [4–6]. This view has been reaffirmed by several public health authorities including Public Health England, The American Cancer Society and the US National Academies of Science, Engineering and Medicine [4–8]. In addition, several organisations have endorsed the use of EC as a smoking cessation aid [5, 7–9] and recognise the role that EC can play in tobacco harm reduction strategies.

Recently, the Centers for Disease Control and Prevention (CDC) estimated that the prevalence of current EC use amongst US adults is between 2.8 and 3.5% [10, 11], the majority of whom are reported to be either “current” or “former” smokers, who use EC “every day” or “some days” [11–13]. The rise in EC use has been associated with the first statistically significant increase in smoking cessation rates among US adult smokers at the population level, after 15 years of cessation rate stagnation [14, 15]. The prevalence of current cigarette smoking amongst the US adult population has declined from 24.7% in 1997 to 14.4% in 2017 [16, 17] and whilst cigarettes are still the most commonly used nicotine product in the US, smoking prevalence is at the lowest levels recorded since the National Health Interview Survey started collecting data in 1965 [10].

To reduce the health burden at a population level, harm reduction policies focus on well-established public health concepts which concentrate on modifying, rather than, eliminating behaviours. The principle of tobacco harm reduction recognises that many adults continue to smoke, despite the implementation of strong tobacco controls and awareness of the associated health risks. It is possible for EC to have a positive and legitimate role to play in public health, particularly among those adults who are uninterested or unwilling to quit smoking, or in those who do not find traditional nicotine replacement therapies (NRT) effective, especially as EC are widely accepted by adult smokers and are a viable alternative to smoking [5, 18]. Adult smokers commonly report using EC to reduce or replace their smoking [19–21] and EC use is associated with a reduction in cigarette consumption [22, 23]. Researchers have reported that whilst some adult smokers who use EC may occasionally have a smoking lapse, EC use does not lead to a full relapse back to cigarettes, suggesting that EC can prevent long-term relapse [24]. Recent clinical research indicates that EC are almost twice as effective at helping adult smokers quit cigarettes compared to medically licensed NRT [25]. Notably, recent data from the UK smoking toolkit reports that EC surpassed over-the-counter NRT in 2013 as the most common product used in quit attempts and has remained the most popular to date [26]. This is especially noteworthy considering that in many countries NRT products are available only with a prescription.

Despite the fact that one of the most commonly cited reasons that adult smokers choose to use EC is to reduce risk to their health [19–21], a growing body of research suggests that US adults lack awareness about the potential relative reduced harm of EC, and increasingly hold beliefs that these products are either as harmful as cigarettes, or more concerningly, that they are worse than smoking [27–32]. Previous research has demonstrated that safety and health concerns are a prominent reason given by adult smokers for either not trying EC [32] or stopping EC use, and returning to smoking cigarettes [33]. In addition, recent work by Brose and colleagues report that perceived relative harm of EC predicted subsequent use in current and former smokers [28]. Therefore, adult smokers who are poorly informed, or hold inaccurate beliefs or misconceptions about the relative harm of EC may be deterred from trying these products, potentially resulting in sustained smoking or relapse to smoking following a quit attempt. An accurate perception of the relative harm of EC is therefore critical in motivating adult smokers to try, and remain, using these products over exclusive lifelong cigarette use. The potential contribution that EC can have on public health at the population level can only be realised if the adult smoking population is clear on the relative risk of EC compared to smoking. However, little is known about how the overall perception of EC risk is evolving over time in US adults. To quantify this, we conducted an analysis of the first three waves of longitudinal data from the US Population Assessment of Tobacco and Health (PATH) study.

## Method

### Data source and sample

The Population Assessment for Tobacco and Health (PATH) study is a nationally representative longitudinal cohort study conducted by the National Institute on Drug Abuse and the FDA’s Center for Tobacco Products. The first wave (September 2013) contains baseline information for the study population and continuing follow-up data becomes available as the consortium collects subsequent waves of data. The PATH study uses audio computer-assisted self-interviews available in English and Spanish to collect information on tobacco use patterns and associated health behaviors. Address-based area probability sampling with an in-person household screening procedure was used to recruit participants. PATH study data collection involves a rigorous, multi-level sampling and a weighting scheme to ensure that data are representative nationwide and adjusted for oversampling and nonresponse. Weighted estimates produced by the PATH study are representative of the non-institutionalised, US civilian population [34]. We used data from the first three waves of the PATH study (2013 to 2016) which were publicly available at the time of this publication. Wave 1 (September 2013–December 2014), Wave 2 (October 2014–October 2015) and Wave 3 (October 2015–2016) adult interviews were analysed in this study.

To assess the trend in perceived relative harm among US adults, we selected individuals who participated as adults at the first wave and who attended the next two waves. Individuals who were unaware about EC at Wave 1 were excluded in this study. In total, this study focused on 21,693 US adults.

### Measure of perceived relative harm

Perceived harm of EC relative to cigarettes was assessed in each wave of the PATH study by a single question (AE1099) which differed between Waves 1 and 2 and Wave 3. During Waves 1 and 2 question, AE1099 focused on EC specifically, "Is using e-cigarettes less harmful, about the same, or more harmful than smoking cigarettes?". During Wave 3, the question AE1099 expanded to “Is using e-cigarettes or other electronic nicotine products less harmful, about the same, or more harmful than smoking cigarettes?”. Five relevant response categories were constructed based on respondents answer to AE1099; “1, less harmful”, “2, about the same”, “3, more harmful”, “− 8, do not know”, “− 7, refused”. Respondents who had missing or incomplete responses for this key measure were dropped from the analysis. Respondents were further categorised into two response categories: individuals who answered, “less harmful” and those who answered “same or more harmful” in response to question AE1099.

### Smoking and vaping status

The smoking and vaping status for 21,693 US adults was assigned according to the definitions given in the PATH study [34] as either “current smokers” or “current vapers”, “former smokers” or “former vapers” or “never smokers” or “never vapers”. For clarity, adult smokers or vapers who were considered as “current experimental” and “current established” were regrouped as “current smokers” or “current vaper” and “former experimental” and “former established” were regrouped as “former smokers” and “former vapers” respectively.

### Future trends in relative harm perception amongst US adult smokers

**Fig. 1:**
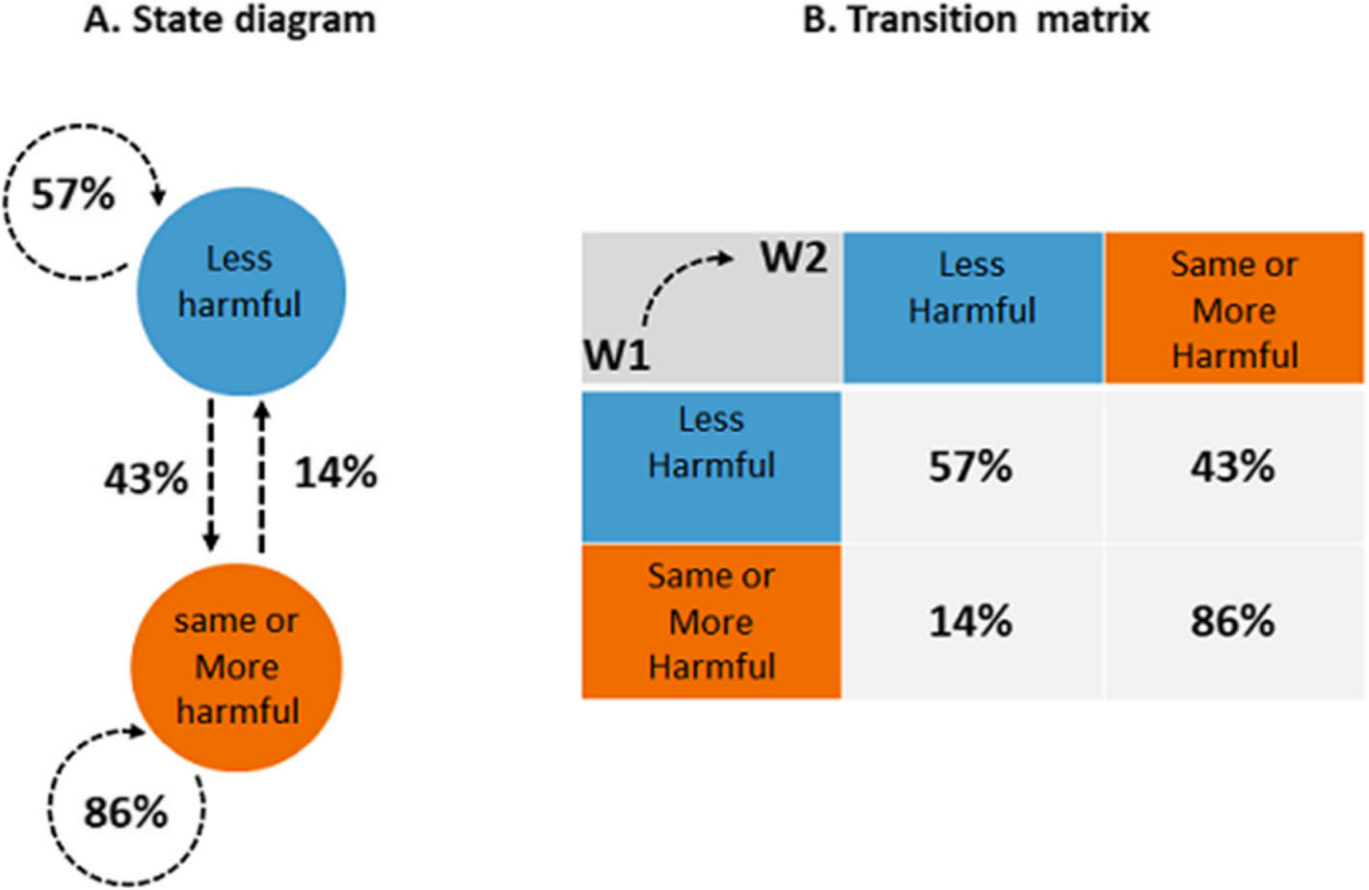
State diagram and the associated transition matrix from Wave 1 to Wave 2. **a** Harm perception state diagram. **b** The associated transition matrix from Wave 1 to Wave 2

To estimate future trends in relative harm perception amongst US adult smokers, we created a model based on the changes in relative harm perception observed amongst the smoking population between Wave 1 and Wave 2. A matrix of transitions and the corresponding state diagram using a directed graph to picture the state transitions was built. Figure 1 represents the state diagram and the transition matrix. The states represent whether adult smokers believe EC are “less harmful than conventional cigarette” or if EC “is about same or more harmful than conventional cigarette”. These two states have been renamed respectively “less harmful” and “same or more harmful”.

A discrete time Markov chain [35] was applied to model the transition probabilities between discrete states and to forecast the prevalence of US smokers who would perceive EC the “same or more harmful than conventional cigarette” if effective intervention is not implemented to correct misconceptions within the adult smoking population.

### Statistical analysis

First, we presented descriptive statistics of the study demographic and analysed cigarette and EC status. The proportions of responses about perceived harm relative to cigarettes were analysed across all three waves. The trends in perceived harm from Wave 1 to Wave 3 for all selected adults were plotted with 95% confidence intervals (CI) for the response category proportions for relative perceived harm. Finally, the impact of smoking and vaping status on relative harm perception trends were investigated. All analyses were conducted using R version 3.5.1 R Core Team. We used chi-square statistical analysis to assess changes in perceived relative harm to investigate whether negative relative harm perceptions amongst former smokers and current vapers at Wave 2 was associated with a return to smoking at Wave 3. The level of statistical significance was set at *p* < 0.05.

## Results

**Fig. 2:**
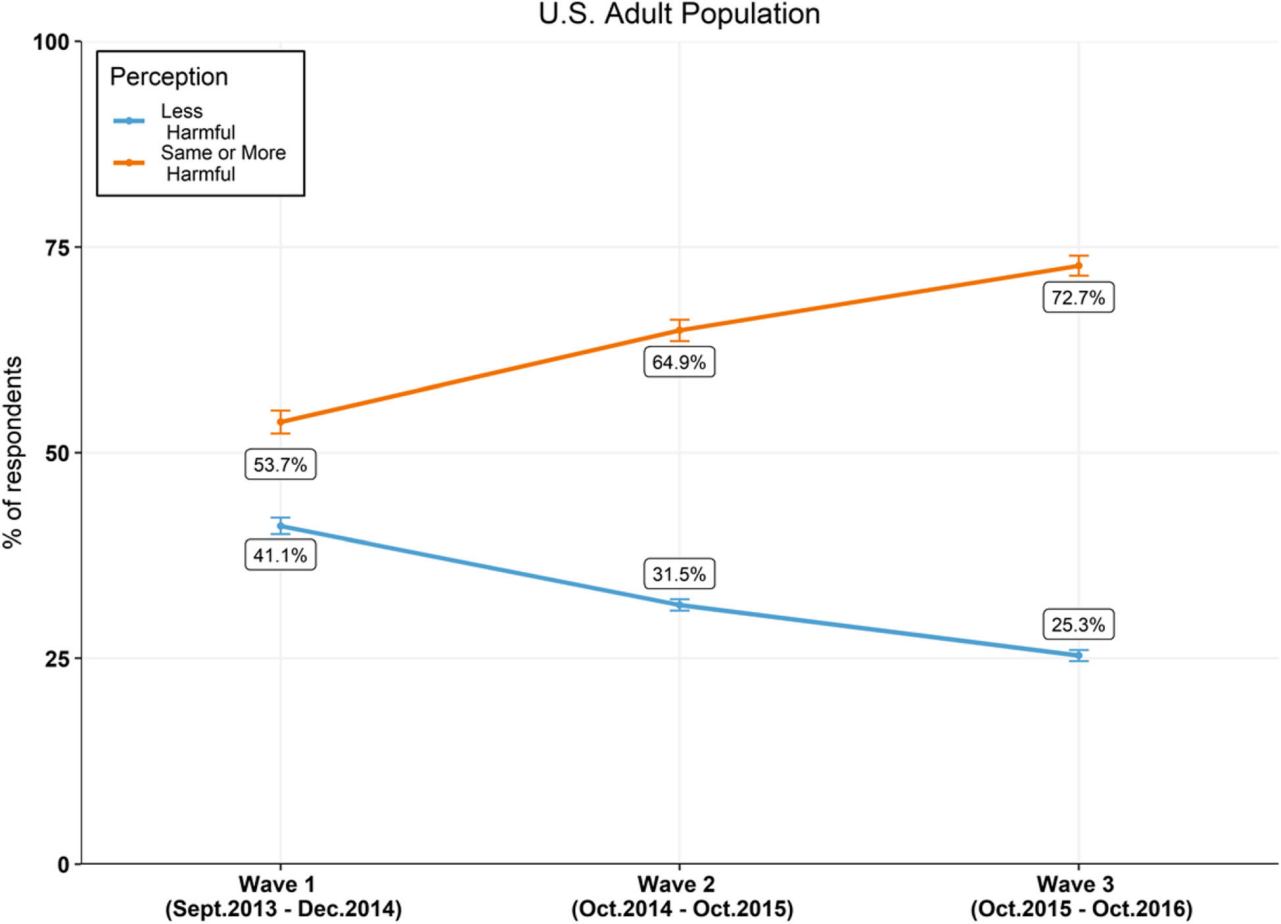
US adult population perceived harm of EC relative to cigarettes between 2013 and 2016. Percentages are weighted. Error bars indicate 95% CI

The proportion of US adults who perceived EC as less harmful than combustible cigarettes has consistently declined each year from 41.1% (CI 40.1–42.1%) in Wave 1 (W1), 31.5% (CI 30.8–32.2%) in Wave 2 (W2) and 25.3% (CI 24.6–26.0%) Wave 3 (W3) (Table 1). Concurrently, during the same time frame, the proportion of the US adult population believing that EC were as harmful or more harmful than smoking combustible cigarettes increased from 53.7% (CI 52.3–55.1%), to 64.9% (CI 63.6–66.2%) to 72.7% (CI 71.5–73.9%) between Waves 1, 2 and 3 respectively (Fig. 2).

**Table 1:** Weighted prevalence for gender, ethnicity, age and perceived relative harm for across all three waves

Characteristics	Modalities	Wave 1	Wave 2	Wave 3
Sex	1 = Male	47.9%	48.7%	49.1%
Sex	2 = Female	52.1%	51.3%	50.9%
Race/ethnicity	1 = Non-Hispanic White	68.3%	68.2%	68.3%
Race/Ethnicity	2 = Non-Hispanic Black	10.9%	10.7%	10.6%
	3 = Non-Hispanic other	6.5%	6.7%	6.8%
Race/Ethnicity	4 = Hispanic	11.1%	11.1%	11.1 %
Category of age	1 = 18 to 24 years old	13.5%	11.8%	9.9%
Category of Age	2 = 25 to 34 years old	17.9%	18.5%	18.7%
Category of Age	3 = 35 to 44 years old	17%	17%	16.9%
Category of Age	4 = 45 to 54 years old	18.8%	17.9%	17.9%
Category of Age	5 = 55 to 64 years old	17.4%	18.1%	18.3%
Category of Age	6 = 65 to 74 years old	10.7%	11.5%	12.3%
Category of Age	7 = 75 years old or older	4.7%	5.3%	6%
Relative harm perception	− 8 = Do not know	5%	3.4%	1.8%
Risk Perception	− 7 = Refused	0.2%	0.2%	0.1%
Risk Perception	1 = Less harmful	41.1%	31.5%	25.3%
Risk Perception	2 = About the same	47.2%	55.3%	62.9%
Risk Perception	3 = More harmful	6.5%	9.6%	9.8%

Race/ethnicity was categorized as white non-Hispanic, black non-Hispanic, non-Hispanic other (i.e. American Indian/Alaskan Natives, Native Hawaiian, Pacific Islander, Multi-racial) and Hispanic of any race. Gender categories included male and female. Ages were categorized into the following groups: 18–24, 25–34, 35–44, 45–54, 55–64, 65–74 and 75 and older. Categories as defined by the PATH study

When smoking and vaping status across all three waves was analysed (Table 2), current non-smoker non-vaper (including former or never users) was the most prevalent status in each wave, 76.0% (W1), 69.5% (W2) and 62.4% (W3), followed by never users of either products 28.5%, 27.8% and 26.8% at Waves 1, 2 and 3, respectively. In contrast, dual use of both cigarettes and EC remained low, reducing across the waves from 4.9%, 4.4% to 3.3% at Waves 1, 2 and 3, respectively. Overall, US adult never users of cigarettes who currently use EC (NVR; CUR) was nominally reported in 0.1–0.2% of the population between Waves 1 and 3.

**Table 2:** Weighted prevalence of smoking and vaping status for all three waves

Cigarette; EC	Wave 1	Wave 2	Wave 3
___ ; ___	0.0%	0.1%	0.7%
___; CUR	0.0%	0.0%	0.1%
___;FMR	0.0%	0.2%	0.4%
___;NVR	0.4%	4.4%	10.2%
CUR;___	0.0%	2.7%	3.7%
CUR;CUR	4.9%	4.4%	3.3%
CUR;FMR	8.6%	8.2%	8.6%
CUR;NVR	8.7%	6.5%	5.5%
FMR;___	0.0%	1.8%	2.6%
FMR;CUR	1.2%	1.6%	1.7%
FMR;FMR	4.5%	5%	5.3%
FMR;NVR	42.5%	36.2%	29.5%
NVR;___	0%	0.3%	0.6%
NVR;CUR	0.1%	0.1%	0.2%
NVR;FMR	0.5%	0.5%	0.8%
NVR;NVR	28.5%	27.8%	26.8%

A small proportion of respondents have at least one undefined status for vaping and/or smoking. CUR corresponds to current smoker or vapers, including “current experimental” (defined as Wave 3 **a**dult respondents who have not smoked at least 100 cigarettes in their lifetime, and currently smoke every day or some days or have ever used an EC, have never used fairly regularly, and uses every day or some days) and “current established” (defined as Wave 3 **a**dult respondents who have smoked at least 100 cigarettes in their lifetime, and currently smoke every day or some days or have ever used an EC, have used fairly regularly, and uses every day or some days). NVR corresponds to never smoker or vapers (defined as never smoked a cigarette, even one or two puffs or never used an EC, even once or twice) FMR corresponds to former smoker or vapers including “former experimental” (defined as ever smoked a cigarette, has not smoked more than 100 cigarettes in lifetime, and now does not smoke at all or have ever used an EC, have never used fairly regularly, and does not use at all) and “former established” (defined as ever smoked a cigarette, has smoked more than 100 cigarettes in lifetime, and now does not smoke at all or have ever used an EC, has used fairly regularly, and currently does not use at all). ___ means that the status has not been defined

**Fig. 3:**
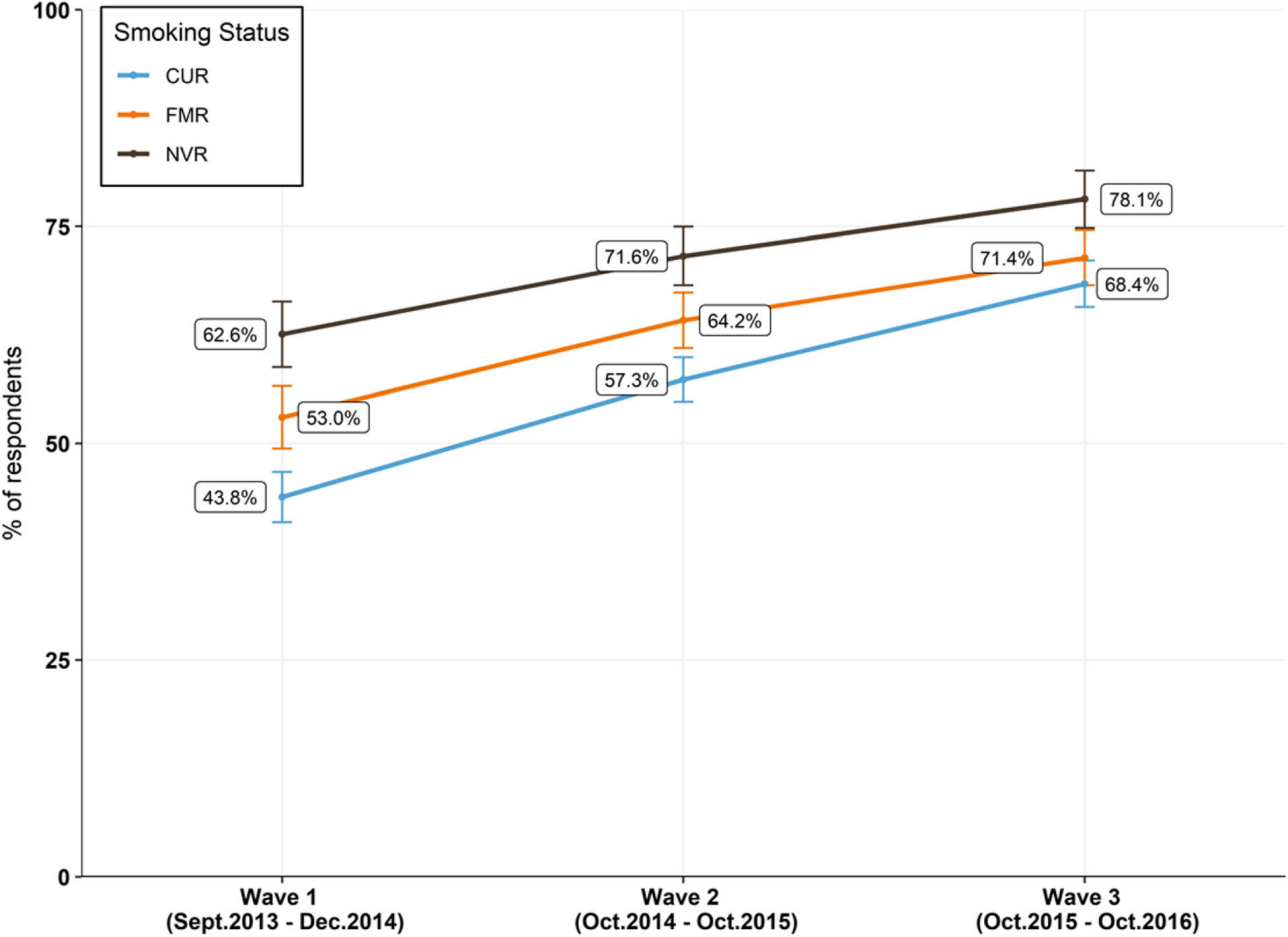
US adults who perceived EC as equally or more harmful than cigarettes and smoking status. US adults who perceived EC as about the same, or more harmful, than cigarettes across all three waves, stratified by smoking status. “CUR” corresponds to current smoker or vapers, “FMR” corresponds to former smoker or vapers and “NVR” corresponds to never smoker or vapers. Percentages are weighted. Error bars indicate 95% CI

Regardless of smoking status, the proportion of adult smokers who believed EC were equally or more harmful than cigarettes increased substantially between 2013 and 2016 (Fig. 3). Among current adult smokers, the percentage of those that held an incorrect relative harm perception increased across all waves by a total of 24.6% percentage points in 3 years, from 43.8% (CI 40.9–46.7%) in W1 to 68.4% (CI 65.7–71.1%) in W3.

**Fig. 4:**
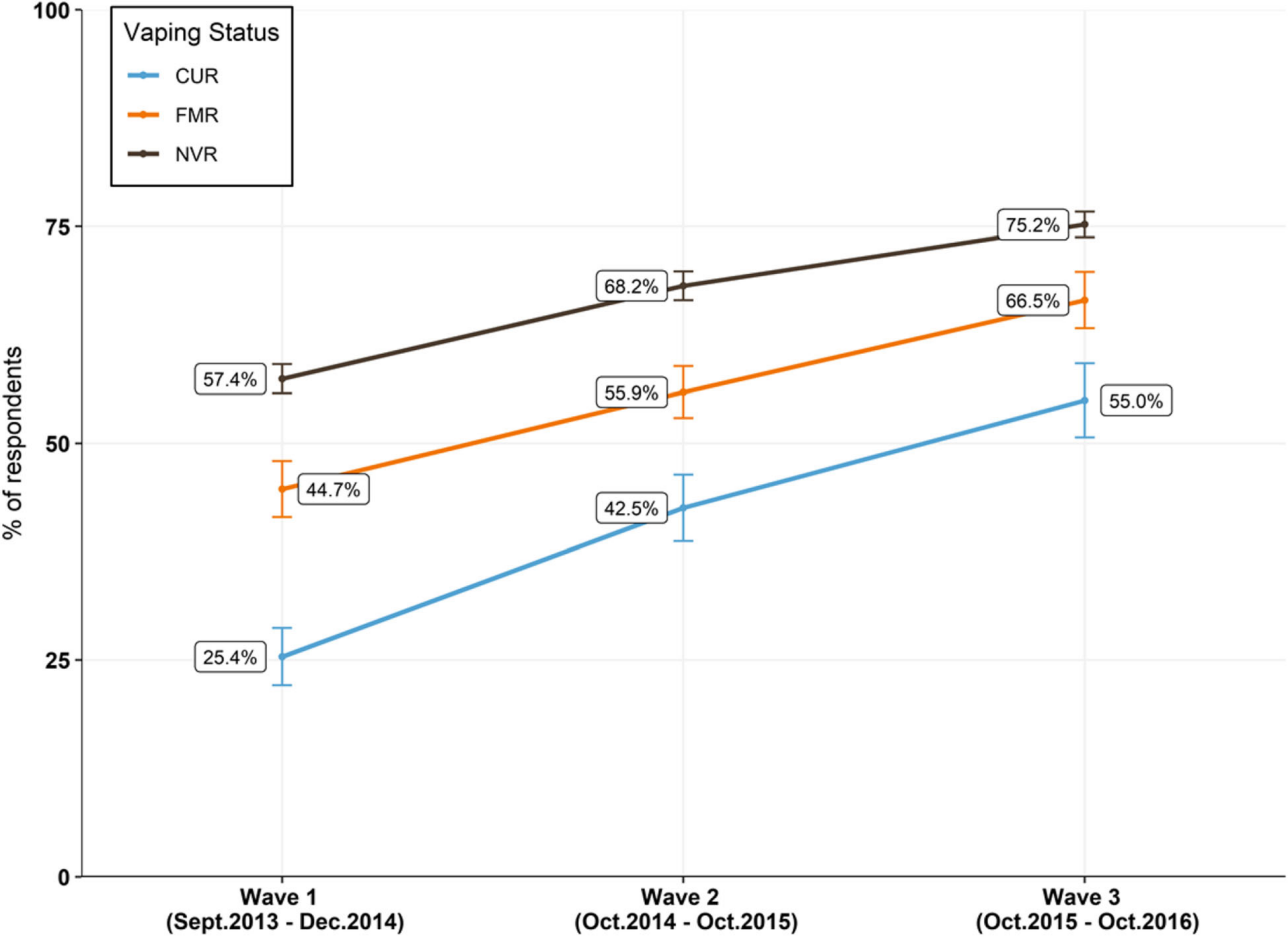
US adults who perceived EC as equally or more harmful than cigarettes and vaping status. US adults who perceived EC as equal to, or more, harmful than cigarettes across all three waves, stratified by vaping status. “CUR” corresponds to current smoker or vapers, “FMR” corresponds to former smoker or vapers and “NVR” corresponds to never smoker or vapers. Percentages are weighted. Error bars indicate 95% CI

To further understand the relationship between perceived relative harm and product use, perceived harm and vaping status was investigated. Similar to what was observed with smokers, regardless of vaping status, the proportion of US adults who perceived the relative harm of EC equally to, or more harmful than, smoking increased substantially across all three waves (Fig. 4). The percentage of current adult EC users who perceived the relative harm of EC equal to, or more harmful than, cigarettes increased by 29.6% over time, from 25.4% (CI 22.1–28.7%) in W1 to 55.0% (CI 50.7–59.2%) in W3. Compared to smoking status, a wider distribution in relative harm perception was observed when data was stratified by vaping status. Overall, a higher proportion of current adult EC users perceived EC as less harmful than cigarettes (43.5%, CI 40.3–46.7%) compared to current smokers (29.3%, CI 28.2–30.4%), never vapers (22.7%, CI 21.9–23.5%) or never smokers (20.8%, CI 19.4–22.2%) in W3.

**Fig. 5:**
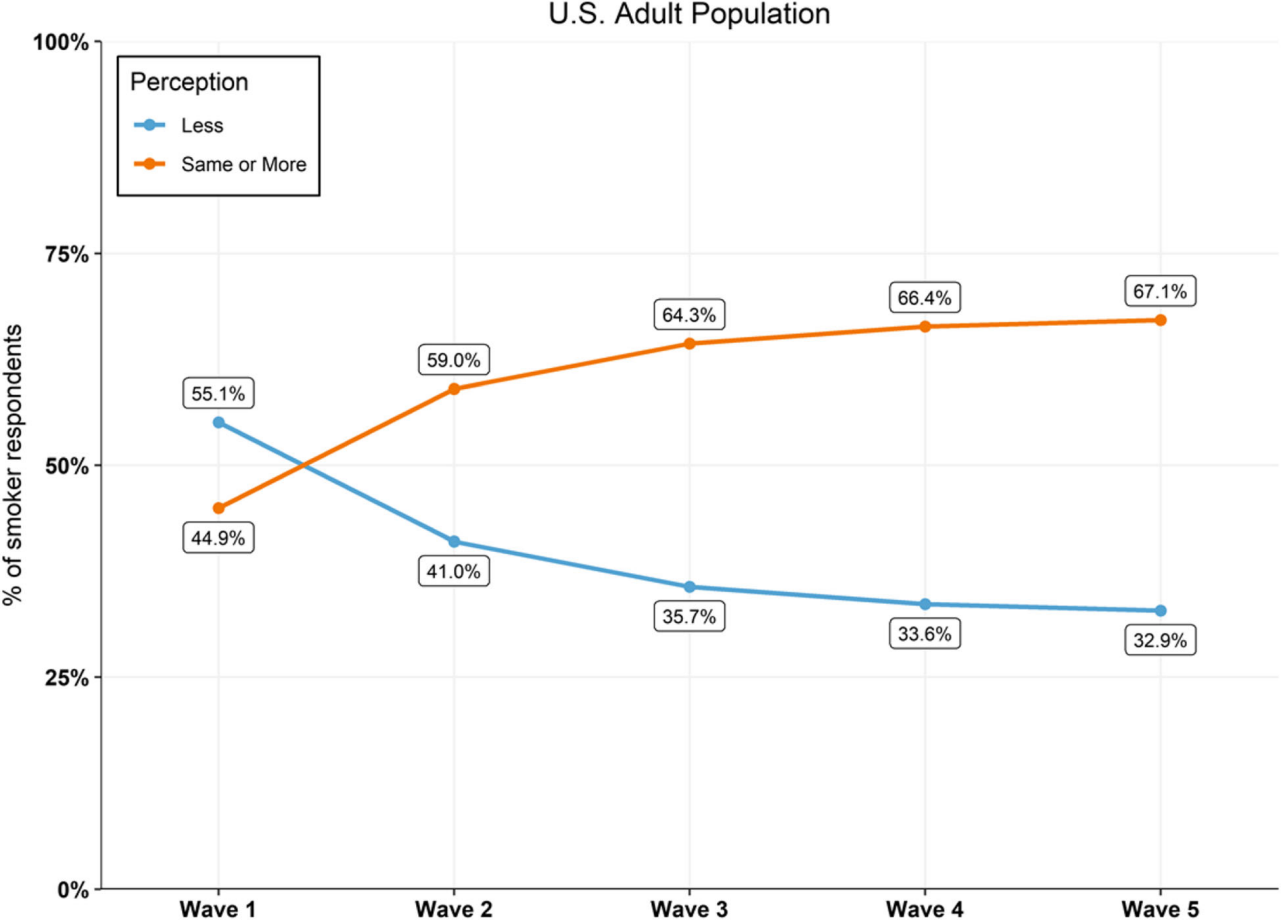
Prediction model estimates for relative harm perceptions amongst US adult smokers

To estimate future trends in relative harm perception amongst US adult smokers, a model was constructed and validated by retrospectively comparing estimated percentages against published PATH data. For Wave 3, the model estimated that 64.3% of US adult smokers would report EC as being equal to, or more harmful, than cigarettes (Fig. 5), a difference of 4.1% percentage points compared to the PATH data (68.4%). When the analysis was expanded to project future relative perceptions if the current trend continues, the model estimated that the percentage of US adult smokers who would incorrectly perceive the relative harm of EC would increase to 67.1% by Wave 5. As the model appears to underestimate the trend in inaccurate perceptions among US adult smokers, it is likely to expect that by Wave 5 the percentage of adult smokers who report incorrect relative harm perceptions of EC would exceed 70% if the trend continues.

Next, we focused on former smokers who used EC in Wave 2 and estimated the weighted proportions of individuals who reported smoking relapse according their relative harm perception in Wave 3. Former smokers who reported using EC at Wave 2 had significantly higher rates of smoking relapse at Wave 3 if they perceived EC as being equal to, or more harmful, relative to cigarettes. Table 3, (29% and 37%, respectively, *p* < 2.2e−16) compared to those who perceived EC as relatively less harmful, where relapse was reported at 19%.

**Table 3:** Smoking relapse between Waves 2 and 3 according to relative harm perception in former smokers

Relative harm perception	Still former smoker	Relapse smoking	% relapse
1 = less harmful	2004002	462707	19
2 = about the same	696429	282810	29
3 = more harmful	81604	47060	37

## Discussion

Our findings confirm that despite many health organisations and regulators adopting a strategy which encourages adult smokers to transition to EC if they are uninterested or unwilling to quit smoking, there is widespread confusion and misconception about the actual relative harm of EC among US adults. Four principle findings emerged from this study. First, analysing data from a nationally representative longitudinal study demonstrates that the proportion of US adults who perceive EC as less harmful than cigarettes decreased substantially between 2013 and 2016. Second, during the same time period, the proportion of US adults who perceived EC to be equally or more harmful than cigarettes increased substantially. Third, compared to ‘never’, ‘former’ or ‘current’ smokers, a lower proportion of current EC users perceived EC equally or more harmful than cigarettes. However, even amongst current users the proportion who perceived the relative harm of EC equal to or more harmful than cigarettes increased between 2013 and 2016. Fourth, former smokers who used EC were more likely to report smoking relapse if they held negative relative harm perceptions compared to those who correctly perceived the relative harm of EC.

Our finding that most US adults perceive the relative harm of EC as equally or more harmful than cigarettes is consistent with findings from several previous studies across multiple countries [27–32, 36]. Our findings do, however, differ from a recent literature review [37] which concluded that the majority of study respondents perceived the relative harm of EC as less harmful than cigarettes. Importantly, this publication reviewed literature up until 2014, and whilst the increase in popularity of EC with adult smokers became noticeable in 2010, a greater increase in use was not observed until 2014. In addition, our findings show that current adult smokers are less likely than current EC users to perceive EC as less harmful than combustible cigarettes. Our study supports others who have reported that relative harm perception of EC compared to cigarettes has deteriorated in more recent years [27, 29, 38], particularly among adult smokers who may benefit most by transitioning to EC rather than continued smoking [39]. Furthermore, our model suggests that inaccurate misperceptions amongst adult smokers will continue to deteriorate unless effective intervention is implemented.

The growing misperception of the relative harm of EC among the adult smoking population may in fact be deterring adult smokers from even trying or continuing the use of these products. Negative beliefs and the further upward trend of perceiving EC as equally or more harmful than cigarettes in Wave 3 warrants heightened consideration and should be investigated further. This is especially critical considering that one of the most commonly cited reasons why adult smokers report using EC is to reduce the impact on their health and to either reduce or replace their smoking [19–21]. Our findings that negative beliefs of EC relative harm was associated with smoking relapse support others who report that positive perceptions of the relative harm of EC is associated with subsequent use in smokers and ex-smokers [19, 28, 40] and is a strong predictor of exclusive use among adult smokers who completely transition from cigarettes [41].

How the relative harm of these products is communicated to the public, and adult smokers more specifically, is critical and requires unique consideration. Studies have found that potential health warnings may inadvertently deter smokers from initiating EC use and that messages conveying reduced harm may be more effective [42, 43].

In addition, it has been reported that statements which do not differentiate relative risks are misinterpreted by consumers to mean that EC are just as harmful as cigarettes [44]. Therefore, effective public communication which accurately interprets the scientific data and clearly differentiates the absolute from the relative risk of EC will increase consumers’ knowledge of the risk differential. Correctly understanding the relative harms of EC could potentially create the health incentive required for those adult smokers who wish to transition for health reasons. Information based on comparative harm is commonplace for many harm reduction strategies; however, tobacco use appears to be exempt from this approach in many countries [45]. Whilst there has been some concern that providing adult smokers with less harmful nicotine-containing products may obstruct smoking cessation, the use of snus in Sweden, a less harmful alternative to cigarettes [46], appears to have facilitated record low smoking prevalence and the lowest levels of tobacco-related mortality amongst men in Europe [47]. The example of snus in Sweden strongly suggests that reduced harm products can positively impact the smoking population. Some researchers have voiced criticism that the dominant public health messages continue to focus on the absolute risk of using any tobacco product rather than the relative risk between categories of products that have the potential to reduce risk and cigarettes [45]. For example, it has been reported that major US health information websites have either stated that smokeless tobacco carried the same risks as cigarettes [48] or made modest efforts to inform consumers on the significantly lower risk of these types of products compared to continued lifelong cigarette use [45]. Health agencies and regulators globally should aim to provide adult smokers with clear, objective and evidence-based health information on the differential risks between using cigarettes and other products. Such information is necessary for individuals to be able to exercise personal autonomy and make informed decisions on which product to use to either reduce or replace their cigarette consumption. Accurate communication of the risk differentials should not be underestimated considering that consumers primarily trust information on the health risks and benefits of EC from prominent public health agencies over other sources [49, 50]. Consequently, information provided by regulators, health agencies and independent scientists about harm reduction may be directly influencing the populations harm perception of EC.

Misconceptions of the relative harm of EC may also be being driven by misunderstandings of the harm of nicotine use and the wider impact of negative media coverage [39]. Inaccurate knowledge of nicotine, and the misattribution of the portion of risk in smoking to nicotine, may have indirectly contributed to the misperceptions surrounding EC [51]. Furthermore, media coverage which reports that EC and e-liquids are linked to toxicant exposure [52], health-related problems [53, 54] or serious injury [55, 56] are frequent. In addition, the absence of a strong distinction between absolute and relative risk may be contributing to a communication bias where media reports are overstating the absolute harm and not contextualising relative risk against continued smoking. That said, it is important that the public are fully informed that ECs are not risk-free, are a harm reduction tool for adult smokers who would otherwise continue to smoke combustible cigarettes and should never be used by non-smokers or vulnerable populations. Moreover, there is a need to fully understand the potential absolute harms of ECs. As evidenced by the recent outbreaks of e-cigarette, or vaping, product use-associated lung injury (EVALI) in the USA, associated with the addition of the thickening agent vitamin E acetate to illicit tetrahydrocannabinol (THC)-containing vaping devices, ECs must only be used as intended by the manufacturer and not adulterated, tampered with or modified with other substances, which may introduce novel EC harms.

Our study has a number of limitations and the data should be viewed in the context of these. In particular, the PATH study was not originally designed to assess relative harm perception in the US adult population, and the current study design only provides a single item for such assessments which may somewhat limit the reliability of the findings. Furthermore, the single question used to assess perceived relative harm differed in Wave 3 from Waves 1 and 2. It is important to note that the expansion of the question during Wave 3 encompasses other electronic nicotine delivery products which could be interpreted as including other products such as heated tobacco, electronic pipe and water pipe devices. This variation in wording may influence comparability across data waves. In addition, the generic question in the PATH survey to assess relative harm may not have captured various aspects of harm on the perceived health risks associated with use. Assessing relative perceived harm of EC associated with specific health endpoints (e.g. heart or lung disease) may result in different harm perceptions compared to overall harm perceptions in general. It is important to also note that due to the design of the PATH study and the availability of the associated public use files, only data from Wave 1 (September 2013–December 2014), Wave 2 (October 2014–October 2015), Wave 3 (October 2015–October 2016) was available for analysis. Given the significant time lag in data availability, an analysis of more recent EC perceptions was not possible. Future work will include an analysis of the public use files for Wave 4 (December 2016–January 2018) when released to determine if EC perceptions have improved or deteriorated further. Furthermore, given the timings and public availability of the PATH data, it was not possible to analysis any potential association between the recent outbreak of EVALI in the USA and EC perceptions, which may have resulted in an even greater change. The outbreak of EVALI, which received extended news coverage worldwide, was associated with the inhalation of the thickening agent vitamin E acetate added to illicit tetrahydrocannabinol (THC)-containing vaping devices; however, news reports often failed to distinguish these important facts from regular nicotine-containing ECs. Consequently, post-EVALI, this may have increased confusion about the relative harms of nicotine-containing ECs, and vaping as an alternative to smoking more generally, which will not be apparent until future waves of the PATH study have been published and data analysed. Those limitations notwithstanding, the findings from this study are consistent with others who have found that the relative harm perception of EC compared to cigarettes is continuing to deteriorate over time.

## Conclusion

The lack of accurate and consistent messaging from both public health agencies and the media may be contributing to public, and more specifically adult smokers’, perceptions about the relative risk of nicotine when decoupled from combustion and tobacco smoke. Until the advent of new nicotine products such as EC, nicotine and tobacco had frequently been seen as indistinguishable in terms of harm. Our findings show that overtime, perceptions of the relative harmfulness of ECs has continued to deteriorate and diverge from the scientific evidence. This highlights an urgency to communicate the relative risks of nicotine and EC use to the public, especially to adult smokers who are unwilling or uninterested in quitting smoking and have not even tried EC, but who would gain the most in terms of a potential health benefit if they did. Whilst deterring non-smokers is a critical objective of EC health messaging, this goal needs to be carefully balanced so that messaging is compelling enough to prevent any unintended consequences of reducing the appeal or minimising the health incentive of EC to those adult smokers who may be considering and motivated to transition. Confusion may potentially be discouraging adult smokers from using alternative, less hazardous products which may ultimately result in a missed opportunity to positively impact health at both an individual and population level. For the harm reduction potential of EC to be fully realised, the relative risks of using these products, compared to cigarettes, must be evidence based and clearly communicated to the public from several trusted sources. Ensuring that the risk differential is unambiguously understood and accepted as accurate by those who currently use combustible tobacco will be critical. Misconceptions need to be urgently addressed with a need for corrective communications by public health bodies to address the confusions created by previously unclear and contradictory EC messaging. Future research and policy-makers should aim to elucidate why the relative harm of EC compared to smoking is widely misunderstood and continues to deteriorate.

## Data Availability

The dataset(s) supporting the conclusions of this article are available in the Population Assessment of Tobacco and Health (PATH) study [USA] Public-Use Files (ICPSR 36498) repository, https://www.icpsr.umich.edu/icpsrweb/NAHDAP/studies/36498.

## Abbreviations

EC: Electronic cigarette
PATH: Population Assessment of Tobacco and Health
NRT: Nicotine replacement therapy
